# Supplementary material for: Combined multiplex panel test results are a poor estimate of disease prevalence without adjustment for test error
Source: PLoS Comput Biol. 2024 Apr 26;20(4):e1012062. doi: 10.1371/journal.pcbi.1012062 (PMC11078360; doi:10.1371/journal.pcbi.1012062)
Supplement: Attachment — Submitted filename: response.pdf [file pcbi.1012062.s007.pdf]

Bristol Vaccine Centre
Level 6
Education and Research Centre
Upper Maudlin Street
Bristol
BS2 8AE

PLOS Computational Biology
PLOS
Nine Hills Road
Cambridge, CB2 1GE
United Kingdom

19 March 2024

Dear Editor,

Re: Methods paper: Combined multiplex panel test results are a poor estimate of disease prevalence without adjustment for test error.

Please consider this significantly revised version of this paper for publication in PLOS Computational Biology.

Thank you for considering this paper. The comments from the peer reviewers were very helpful and we have incorporated all their feedback into this revised manuscript. The changes we have made should reassure the reviewers on the points they raise, but we have also responded directly to the points they raised below.

Yours sincerely,

Dr. Robert Challen Ph.D. MBBS

Editors comments

Thank you very much for submitting your manuscript "Combined multiplex panel test results are a poor estimate of disease prevalence without adjustment for test error." for consideration at PLOS Computational Biology.

As with all papers reviewed by the journal, your manuscript was reviewed by members of the editorial board and by several independent reviewers. In light of the reviews (below this email), we would like to invite the resubmission of a significantly-revised version that takes into account the reviewers' comments.

The manuscript has been read by two experts in the field, and more briefly by myself. We all find the manuscript novel and interesting. Still there are some issues to be taken care of before potentially being accepted for publication in PLoS Comp Bio. Please address the good points raised by both referees. In particular you should include your novel finding in the abstract (ref 1) and address the two main concerns of ref 2.

Thank you. We have updated the abstract and made changes throughout the manuscript to address the major concerns raised.

Reviewer 1

This paper is a welcome addition to the literature on diagnostic testing. To the best of my knowledge, it is the first to address the context of multiplex testing when 1 or more out of n tests positive equates to a positive diagnosis. The provision of an R package is a bonus,

I felt that the presentation in the paper itself could be streamlined, given that the Supplementary Materials contain everything necessary for anyone who wants to dig into the details of the method. To this reader, the killer diagram is Figure 4, which very nicely shows the counter-intuitive nature, and hence the practical importance, of the solution to the problem.

Thank you for this encouragement and your helpful comments below. In addressing comments we have been mindful of streamlining the paper, but responding to concerns has necessarily involved some additional content in the main paper. The very technical aspects have been pared down as you suggest below.

Abstract. People who only read the abstract will miss the counter-intuitivity – please add a sentence or two

We have updated the abstract to include this sentence: “In this paper we develop a mathematical framework to characterise this issue, we determine expressions for the sensitivity and specificity of panel tests. <u>In this, we identify a counter-intuitive relationship between panel test sensitivity and disease prevalence that means panel tests become more sensitive as prevalence increases.</u> We present novel statistical methods that adjust for bias and quantify uncertainty in prevalence estimates from panel tests, and use simulations to test these methods.”

Line 27. There's a LaTeX glitch here … "Figure /reffig1 E,"

Fixed, thank you.

Lines 33-34. "we define a multiplex test as consisting of a set of independent components which test different independent hypotheses" This is not so much a definition as an assumption (a word you use later) … and it's critical to everything that follows. It does not help that the word independent is being used in two different senses – independence of results given the true disease state and independence of sub-disease states. I'm not a biologist, but I can imagine circumstances in which test results might not be independent, in either sense. An example would be Kato-Katz tests for the three main species of soil-transmitted helminths. Please call this an assumption and add a sentence or two to the discussion.

Agreed. We have updated lines 31-39 as follows: "In more formal language, we assume a multiplex panel consists of a set of component tests which test different hypotheses, the results of which are combined to give a panel result where a positive test result in any component implies a positive test result in the panel. It is assumed that subtypes of disease are independent, and a second subtype may co-occur in a patient with one subtype, with the same likelihood. We also assume that test errors are also independent and do not correlate either with disease or other test results."

Additionally, to address reviewer 2's comments we have strengthened the discussion of independence considerably with the following acknowledgement of limitations in lines 297-318:

"This analysis assumes independence of the subtype diseases, and that multiple subtypes of disease may be present in the same individual at once. This may not always be the case, for example, in-host competition between disease subtypes is known to occur in dengue infections in mosquitos \cite{pepin2008}, and facilitative co-operation between pathogens is also described \cite{singer2010}. Addressing these kinds of diseases would require a different statistical analysis, and we anticipate panel prevalence estimates that assume independence such as those presented here, will be lower than ones which assume competition, although this will be less obvious at low prevalence. We also make the further assumption that test error is independent. Test errors could become correlated if there is close overlap between the epitope being tested for in one subtype versus another, and this is observed in pneumococcal urine antigen detection (UAD) testing \cite{bonten2015}. In this scenario we expect the major problem to be false positives associated with true positives in a closely related subtype, which could result in overestimates of the prevalence of the offending subtype. However, the combination of a false positive and true positive in a panel is always correctly interpreted as a true positive, so panel prevalence estimates will be less affected. This is a limitation of the method as it stands at the moment and further research is required to characterise this, but we note that data to support this are scarce."

Mathematical analysis and validation section. I think some readers might be unnecessarily put off by your notation, which would be a shame. You acknowledge (but in the previous section) that the equations are "not essential to the remainder of the analysis presented in this summary paper" I understand why you need this level of formality in the Supplementary Materials but here I would be inclined to just give verbal statements of the quantities being defined, or at least try to avoid indicator functions and compound subscripts.

Agreed. We have pruned unnecessary mathematical detail from the main paper and retain only the headline relationships, which are needed to support statements in the discussion. We are expecting PLOS Comp Bio's readership to be technically minded.

Lines 136-137 and S2 "This places a limit on the precision of estimates of component test sensitivity, which in turn makes interpretation of test positivity in both components and panels challenging." Indeed it does, although not (IMHO) for the reason you imply. Probably the best (and certainly the most elegant) way to account for uncertainty in Se and Sp is through a Bayesian analysis, which I would make a bit more prominent in the paper itself. The point does need to be made explicitly somewhere that apart from excluding logically impossible values, the actual test results are uninformative about Se and Sp, whereas they are highly informative about prevalence given Se and Sp. For many pragmatic Bayesians, this justifies using an uninformative prior for prevalence, but raises disquiet about reliance on necessarily informative priors for Se and Sp. However, you assume that you do have control data available (albeit not as much as you would like) so you can these to construct empirical priors. Granted this gives you total of 2n+1 priors, but it does seem defensible to treat these as independent and convert the known Se and Sp result to a Bayesian posterior by numerical integration. Presenting the Bayesian version in this way might be more transparent to the reader than it appeared (to me at least) in S1 and S2.

Agreed. We have bought more detail on the Bayesian methods into the main paper in support of figure 5. We have added the following based on your comment between lines 216-226: "The Bayesian analysis assumes an uninformative prior on the prevalence of components and panel, but test results themselves are uninformative about sensitivity and specificity. On the other hand, test results are highly informative about prevalence given knowledge of the sensitivity and specificity. The Bayesian analysis therefore requires informed priors for sensitivity and specificity, and apart from excluding logically impossible values, does not have enough information to also determine sensitivity and specificity. Disease free control group data informs specificity, which is relatively easy to obtain experimentally. Disease positive control group data can be used to inform sensitivity but is practically harder to obtain, particularly at a component level. For frequentist approaches, control group data can be used to construct empirical sensitivity and specificity estimates."

Lines 219-220. “This is counter-intuitive as test sensitivity is usually regarded as independent of prevalence.” This important message needs to be given more prominence in the abstract and in the main body of the paper.

Agreed. The abstract has been updated as mentioned above.

In the mathematical analysis section between lines 106-115 we include the following explanation of the phenomenon: “There is the possibility that a false negative test result in one component can be ``corrected'' by a false positive result in another component, resulting in a panel result that is correct but for the wrong reason, or otherwise masked by a true positive detection of another co-occurring component. This leads to the panel sensitivity being a complex expression which counter-intuitively depends on the prevalence of the condition it is measuring.”

Also, we have included the following in the discussion section between lines 254-260, to draw out the implications of this finding: “Because of the relationship between panel sensitivity and prevalence, the degree of bias depends on the prevalence we are trying to measure. This makes panel test positivity very hard to interpret, even when the sensitivities and specificities of component tests are unchanged. Panel test positivity from two experiments conducted using identical tests in populations with different true prevalence cannot be compared, nor can any degree of statistical significance be attributed to differences between the populations.”

Lines 225-226. “the expected value of test positivity is not a binomially distributed quantity” Something wrong with the wording here. An expected value doesn’t follow any probability distribution, it’s a function of the parameters of the distribution with respect to which it is calculated. Do you mean empirical prevalence? I guess not, because if patients give independent results then the number of positive results (however defined) does follow a binomial distribution. So what do you mean?

Thank you. We had conflated apparent prevalence, i.e. test positivity, with the count of positive tests in several places in the manuscript and supplementary. What was meant was the following, which is found at lines 249-252: “The count of positive panel tests in a sample is not a binomially distributed quantity, due to false positive and false negative results in the components (see Fig~\ref{fig2}). We cannot use binomial confidence intervals for estimates of panel test positivity.” and this section has been modified to further discuss the counter intuitive nature of the relationship between sensitivity and prevalence as above.

In my response to reviewer 2 you will also notice that we made the same mistake in the mathematical description of the Bayesian model. This has now been corrected.

Reviewer 2

The authors present statistical methods for prevalence estimation in the context of multiplex panel tests, more specifically for the case that the panel is used to test for multiple subtypes

of the same pathogen from which then an overall prevalence or burden of disease is to be calculated. They show that in such a situation, small errors on the subtype test level compound and may result in significant bias of the overall prevalence estimate. The authors developed statistical methods to correct for these biases and used simulations to test their methods. They show that a counter-intuitive aspect of multiplex panel tests is that panel test sensitivity depends on component distribution, component test sensitivity and specificity, and on disease prevalence. As an example for a multiplex panel test, they use pneumococcal pneumonia that can be caused by more than 20 pneumococcal serotypes.

The paper addresses an interesting and important problem, is well structured and written, and cites relevant literature. To my knowledge the approach is novel and could be important for other researchers confronted with the task of prevalence estimation based on panel tests. For such researchers, the authors also provide tools implementing their approach in the form of a freely available R package.

I see two major issues with the paper. One is that the authors sometimes seem to contradict their own assumption of subtype independence. The second is the sensitivity of their correction methods to prior mis-specification which may question the applicability in practice. See below for more detail.

If the authors can address these issues appropriately, I would recommend the manuscript for publication.

Many thanks for both going through this manuscript in such detail, and for your positive overall assessment. We've thought carefully about the major issues you raise. These identified a number of areas of weakness in the manuscript and have resulted in a set of changes that we hope will reassure you, which we describe in detail below. We think your concerns relate more to the description of the methods, than the methods themselves, which we believe to be on solid ground.

Major issue 1: Assumption of independence of subtypes

In the simulations in the supplementary appendix S1 (section 4), the authors seem to assume that subtypes do not occur together so that the subtype prevalences add up to a total prevalence (S1, page 6, line 6). This is in contrast to the assumption of independently occurring subtypes that the panel prevalence estimator is then derived from. Does this affect the validity of the simulations?

The simulation in supplementary appendix 1 originally generated component prevalences using a plain ratio of an overall simulation prevalence, which on the face of it creates components that are mutually exclusive, as you rightly highlight.

This was a shortcut on our part to put the component prevalences in the right ballpark, however, the simulation proceeded to generate synthetic data assuming independence, and summarised those data to get to a measure of true prevalence assuming independence. In the comparisons that followed, estimates of prevalence using test positivity (apparent prevalence) were compared to simulation disease positive rate (which is a per-simulation gold standard for prevalence that does not depend on randomisation). The simulations did not compare prevalence estimates to the target panel prevalence that was set as a

simulation parameter. If they had, there would have been a difference. In summary in response to your point 1a) it did not affect the validity of the simulations. You raise an important point though, and we updated the code to set up the simulations properly, and have re-run all the simulations in S1 as will be described below.

It was clear from this that the documentation of the simulation in S1 was inadequate and we have significantly rewritten this for precision and clarity. The changes are too extensive to describe here but can be seen in the latexdiff of S1. We would greatly value your review of this revised simulation section in S1.

With 20 or more serotypes, even if each of them only occurs with low prevalence, co-infection should occasionally be observed if they are independent. E.g., if we assume independence for 20 subtypes and a prevalence of 0.5% for each subtype, then the sum of the prevalences is 10%. But if we use a binomial probability for prevalence calculation (i.e. taking into account co-infection; in R: `1 – pbinom(0, 20, 0.005)`) then we get an expected (panel) prevalence of 9.54%. On the other hand, if co-infection is never observed in reality, then the serotypes may not occur independently after all, and a different statistical approach would be needed. For pneumococcal pneumonia, is it known whether different serotypes can co-occur in the same patient?

We agree that this is how we expect the independent nature of pneumococcal serotypes to behave. We have added in the following text to lines 123-126: “There are over 100 pneumococcal serotypes, and mixed serotype carriage is common, particularly in children. Commensal pneumococcal serotypes opportunistically cause disease, and during disease episodes, multiple serotypes are occasionally detected when tested for. For this simulation we focus on the 20 serotypes that are covered by the 20-valent polysaccharide capsule vaccine (PCV20).“ to the methods. Are they truly independent? We think so, but in reality we don’t know for sure. Co-infection is detected using the methods described here and so may potentially be the result of test error. Multiple serotypes are seen in upper respiratory tract colonisation (more commonly in children) but when invasive disease occurs, usually only one serotype is detected. Detection in this case however is the result of a sampling process involving culture results which are likely to select out just one serotype. Other methods that can detect multiple do find multiple. As with most things in biology the answer is not clear cut. If one assumes independence then there is the interesting possibility that co-detection rates could inform us of relative test sensitivity and specificity between different serotypes. Co-occurrence numbers are somewhat small to do this but this is an avenue for future investigation.

In this context, is it correct that in Fig. 3 B and Fig. 4 the “true prevalence” is the sum of the component prevalences (thus not taking into account co-infection)? This question also applies to the panel prevalences in Fig. 5 (PCV7 to PCV20).

No, all the figures are generated using true prevalence measures that assume independence. To reassure you on this point we can only point you to the simulation code

(links in footnotes): Theoretical Panel prevalence[1] is always calculated assuming independence using the equations from the text. Simulation panel prevalence[2] was calculated from the component prevalences assuming independence, rather than using a simulation parameter, (usually because it was more convenient to do so in multiple simulations). Simulation disease positive rates[3] were calculated directly from the simulated disease status, as simulation randomness will result in difference between simulation disease positivity and simulation prevalence parameters.

Additionally, we had written "If a condition is composed of many subtypes, then each individual subtype must be a fraction of the overall condition prevalence. The more subtypes in a multiplex panel, the smaller that fraction will be, without loss of generality.", on which you commented, "This assumes that subtypes cannot occur together and seems to contradict the assumption of independent hypotheses stated in the previous paragraph."

Whilst we can see how your interpretation has arisen, the statement in a technical sense applies to both independent and dependent cases. In the independent case we think the limits on the average prevalence of a component are as below:

$$1 - prev_N = \prod_n (1 - prev_n)$$

$$prev_n \leq prev_N$$

$$\frac{prev_N}{|n|} \leq \overline{prev_n} \leq 1 - \sqrt[|n|]{1 - prev_N}$$

And so, if the number of components goes up the average component prevalence must go down (assuming prev_N is less than 1). Obviously the wording of our original statement was a cause of confusion so we have rephrased lines 40-44 as follows:

"If a condition is composed of many subtypes, then the prevalence of each individual subtype must be less than or equal to the overall condition prevalence; and on average the subtype prevalence must be less, even when we account for the possibility of co-infection with multiple subtypes. The more subtypes in a multiplex panel, the smaller that average prevalence will be."

In summary, the assumption of subtype independence warrants more discussion.

---

[1] https://github.com/bristol-vaccine-centre/testerror/blob/main/R/panel_prev.R

[2] https://github.com/bristol-vaccine-centre/testerror/blob/ab2230bcfbc86e0028c189a6a9b60e817b21fb80/vignettes/vignette-utils.R#L304

[3] https://github.com/bristol-vaccine-centre/testerror/blob/ab2230bcfbc86e0028c189a6a9b60e817b21fb80/vignettes/vignette-utils.R#L454

We agree with this and along with the changes mentioned above, we have added into the discussion section a limitations paragraph around independence which is as follows, into lines 297-318:

"This analysis assumes independence of the subtype diseases, and that multiple subtypes of disease may be present in the same individual at once. This may not always be the case, for example in-host competition between disease subtypes is known to occur in dengue infections in mosquitos \cite{pepin2008}, and facilitative co-operation between pathogens is also described \cite{singer2010}. Addressing these kinds of diseases would require a different statistical analysis, and we anticipate panel prevalence estimates that assume independence such as those presented here, will be lower than ones which assume competition. We also make the further assumption that test error is independent. Test errors could become correlated if there is close overlap between the epitope being tested for in one subtype versus another, and this is observed in pneumococcal urine antigen detection (UAD) testing \cite{bonten2015}. In this scenario we expect the major problem to be false positives associated with true positives in a closely related subtype, which could result in overestimates of the prevalence of the offending subtype. However the combination of a false positive and true positive in a panel is always correctly interpreted as a true positive, so panel prevalence estimates will be less affected. This is a limitation of the method as it stands at the moment and further research is required to characterise this, but we note that data to support this are scarce."

Major Issue 2: Mis-specification of priors

As the panel prevalence adjustment method is very sensitive to the specificity of the component tests (see Fig. 4), correct prior assumptions for component sensitivity but especially specificity seem to be very important for the prevalence correction to work (see S2, Fig. 5). Although the authors acknowledge this fact (e.g., in lines 201-205), they describe that the method may fail in the case of "complete mis-specification" (line 247) or "extreme misspecification" (S2, Fig. 5 legend). But can a prior specificity of 99.5% instead of a true value of 97.5% (S2, Fig. 5 C) really be considered an extreme or even complete mis-specification? How practical can the method be with real-world data and real-world test accuracy information in the light of such sensitivity to prior mis-specification?

(N.B. we have assumed in responding to this comment that there was a typo and the reviewer meant to write "... instead of a true value of 99.75% (S2, Fig. 5 C) …" rather than 97.5% which is the value referred to in the comment but which is not present in S2, Fig 5 C.)

Thank you for highlighting this area of concern. We reviewed this in detail and felt that part of the problem is that the terminology we used around misspecification was imprecise. We have updated this, as will be described shortly.

The examples in S2 Fig 5 are based on a simulation with a (synthetic) specificity control group with sample size 800. The difference between 99.75% and 99.5% in such a control group equates to 2 false positives versus 4 false positives. The probability of seeing 4 or more false positives in a control group with a true specificity of 99.75% is 0.15. However the priors are provided for each of the 20 components. Therefore a specificity estimate of 99.5% for all 20 components using the control group could only really be likely as the result of a

systematic bias affecting all 20 components in the same direction, which is very unlikely to occur by chance. We think therefore that although this is not misspecification of the priors, it would be systematic bias in the priors, which results in bias in the adjusted estimates, and have updated the manuscript (lines 290) and supplementary materials S2 to reflect this.

In terms of real world practicality, the ability to correct the panel prevalence bias, and get an accurate central estimate is not the only useful aspect of this work. Another very important benefit is being able to express the uncertainty that the test error generates, and include in that uncertainty of sensitivity and specificity. This allows us to understand whether our experimental set up has sufficient power to draw conclusions or make comparisons. In many situations test specificity is not a fixed quantity as tests that produce continuous outputs can be re-interpreted with different cut-off values selecting operating points on their receiver operator curve, allowing a trade off between sensitivity and specificity. We think the uncertainty propagation in this framework gives us the tools to identify problems and evaluate strategies for optimising test performance in the real world.

We have updated the discussion between lines 283-288 to reference this: "With a poorly understood test it is hard to draw any conclusions from the results of a multiplex panel test, however, the methods presented here remain valuable as a way of detecting when multiplex panel tests are underpowered, as insufficient characterisation of component sensitivity and specificity are exposed in the confidence intervals of modelled true prevalence estimates."

Minor issues

1. *Because the methods (as described in the supplementary files S1 and S2) provide the foundation for the paper, it is important that the details in these files be correct. To my understanding, the mathematical notation contains several mistakes that should be corrected or clarified (see attached supplementary files with comments). Both supplementary files should be diligently proofread by the authors to ensure correctness and consistency.*

Agreed, and please accept both our apologies for the errors, and our gratitude for your efforts reading through the manuscript in such detail. Where you have raised issues which are simple changes we have made the change and not responded to comment directly but all changes we have made can be seen in the latexdiff outputs. Where clarifications were needed or there was a more complicated change needed we have copied your comments into this document and responded more fully below. This round of proofreading has identified a few additional issues beyond those you mention so we hope we are now more correct and consistent. It was clear, reading the comments, that particularly the description of the simulation in supplementary 1 needed improvement. As mentioned above, we have therefore extensively rewritten this.

2. *In Fig. 2, the panels C, F, and I are not explained in the figure legend.*

The text has been updated to clarify and the labelling of the quantities in the figure panels improved. The legend now reads: "Distribution of false positives (cyan bars, with expected value, \(E(FP)\), as a blue vertical line) and false negatives (orange bars, expected value, \(E(FN)\), red line) of 1000 hypothetical test results with 0.9975 specificity and 0.8 sensitivity at different prevalence levels. (A), (D) and (G) show the disaggregated distribution of false

positives and false negatives and (B), (E) and (H) show the combined error distribution of test positive observations (grey bars), and expected test positivity (magenta line, \(E(Test pos)\)) compared to the true condition positives (black line). (C) shows numerically the parameters plotted in (A) and (B); (F) relates to (D) and (E); and (I) to (G) and (H)}"

3. *In Fig. 4, is the "component specificity" axis logarithmically scaled? This should be mentioned.*

It was, in fact, logit scaled and this is now stated.

4. *Fig. 5 could be improved by ordering the components not alphabetically but according to the PCV groups and then also indicating the PCV groups with horizontal brackets below the serotype labels.*

Figure 5 has been updated as suggested (and fig 2 in S2).

5. *A few typos and confusions (specificity --> sensitivity in line 217 on page 9) should be addressed (see attached manuscript file with comments).*

All manuscript typos have been corrected. Many thanks for your detailed examination in this regard.

6. *I failed to install the `testerror` R package from the Bristol Vaccine Centre repository. However, installation of the development version from github worked fine.*

We are glad it worked eventually. If you have any details on the installation failure please raise an issue on Github. We have some small fixes that have arisen to `testerror` and one of the supporting packages (`interfacer`) which we will aim to have released before this reaches you.

Supplementary 1 - inline comments

Latex formatting and minor typographical changes suggested have all been accepted and updated and are not specifically mentioned in this response. A few additional issues were identified and corrected. The changes are visible on the latexdiff.

Have different pneumococcal serotypes been found in the same patient?

Yes, this is now discussed in more detail in the main paper, so we have not changed anything here as S1 does not specifically reference pneumococcal disease.

"However, in practice, this is less of an issue than the Rogan-Gladen estimator, and truncation is not required to produce reasonable estimates of combined sensitivity." Why is that?

Text updated to read: "However, in practice, this is less of an issue than the Rogan-Gladen estimator, and truncation is not required to produce reasonable estimates of combined sensitivity, due to the Rogan-Gladen term (2) being in both numerator and denominator."

"Each simulation consisting of between 2 and 9 components" Is this N_sim?

Yes. We have updated this and made an effort to refer more clearly to model terms in the text throughout.

“constrained so that the total prevalence matches a simulation parameter (prevNsim).” - This assumes that subtypes do not occur together, contrary to the assumptions that the estimator is devired from.

As you will understand this work has been through several iterations. This simulation did use the stated method which does not assume independence to generate component prevalences, but then proceeded to perform the simulation assuming independence and calculated the panel prevalence correctly. The comparisons that were subsequently made in the plots were not to the parameterised prevalence, but the simulation disease positive rate, which was also correctly accounting for independence. In summary the simulation was set-up incorrectly but behaved correctly subsequently. The prevNsim parameter was never used after set-up.

We have updated the code in supplementary 1 to use the same method as the simulation in supplementary 2. All the simulations in supplementary 1 have been re-run and all plots updated. This has not given rise to any major difference in the simulation outcome, because the comparison in the QQ plots is between the simulation disease positive rate and the unadjusted and adjusted test positive rates. There are minor changes in the error plots as a result of re-running the simulations with different randomisation, but these do not affect interpretation.

<table>
<tr><th>New</th><th>Old</th></tr>
<tr><td>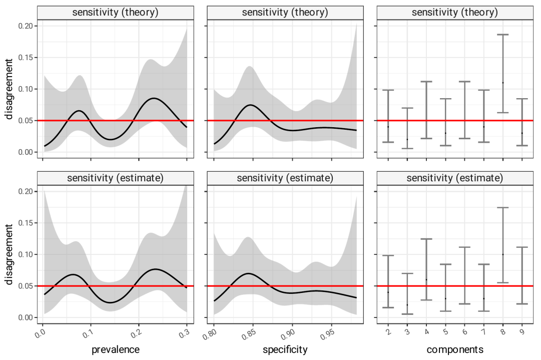
</td><td>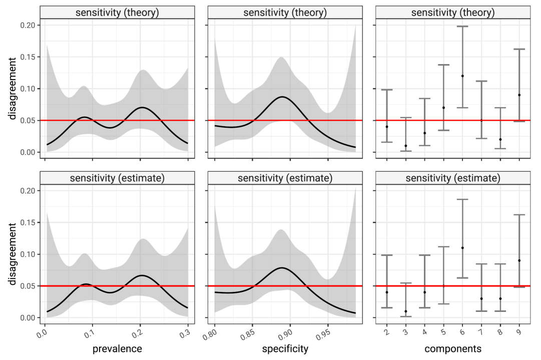
</td></tr>
</table>

What became clear on addressing this comment and others arising in the simulation section in S1 was that the description of the simulations and the nature of the comparisons was inadequate. We have restructured this description, broken it into 2 parts (prevalence validation, and sensitivity and specificity validation) and added in key details to clarify the comparisons being made. Your review of the revised simulation section in S1 would be valued.

“ including confidence intervals,” - Confidence intervals not defined in (9)?

The confidence intervals for simulation-derived sensitivity and specificity estimates are binomial confidence intervals derived from the counts of true positives versus count of positives. This section has been heavily rewritten as per the comment above, and this detail has been clarified. For estimating binomial confidence intervals we used Wilson's method which has been appropriately cited.

"Figure 2: … simulation prevalence" - Is this prev_N from (8)?

As described above, this comparison was actually made using the simulated disease positive rate as a per-simulation gold standard for prevalence. We have made this explicit throughout and we have labelled it throughout as \overline{prev_{N,sim}}. This may not be exactly the same as the simulation parameter, prev_N_sim, depending on the random generation of the simulation. As mentioned above, the difference between these 2 quantities is the reason that the simulation self corrected when the set-up incorrectly assigned non-independent prevalences.

"Figure 4:" - Please add explanation for pink data (presumably estimates where the CI of the estimate does not cover the true value?)

Figure legends for Figs 3 and 4 both updated. Your intuition was correct.

Supplementary 2 - inline comments

Latex formatting and minor typographical changes suggested have all been accepted and updated and are not specifically mentioned in this response. A few additional issues were identified and corrected. The changes are visible on the latex diff.

"Figure 1: " - The black dot denotes the critical prevalence for the assumed sens and spec values? I think it would be preferable to plot the diagonal on top of the blue shading.

Agreed. This was a PDF transparency issue. The figure has been updated and additional clarifications added. The colour scheme has also been changed, resulting in a few changes to the text. The dot is now white.

"… with a single example as shown …" - A single example of a simulated set of patients?

Text is clarified to refer to a "set of simulations", and "One such simulation shown in Fig 2".

" defined prevalence levels, from 2.5% to 20%." - Make clear that this is the panel prevalence for the PCV20 group.

Text updated from "prevalence levels" to "PCV20 panel prevalences".

"The simulation includes k synthetic patients (1000 was used in all cases) …" - This should rather read something like "a set of K synthetic patients (1000 was used in all cases), indexed by k".

Text updated as suggested

"only a restricted number of representative scenarios were tested."- How many were these in total?

In fact this sentence did not really mean much. All scenarios described were tested but there are an infinite number that we could have tested. We have removed this statement.

“\widehat{AP_N}“ - not defined in the model

The definition of this has been added in the simulation on page 4

Multiple comments in Bayesian model section: e.g. “\widehat{AP_n} \sim Binomial(|Ksample |, AP_n).” - factor 1/|K_sample| missing? and That does not belong here, right?

The Bayesian model, unlike the other approaches, uses raw test positive counts rather than apparent prevalence estimates. This was incorrectly labelled in the formulae, which referred to apparent prevalence (\widehat{AP_N}), as did surrounding text. We have made this distinction clear and now refer to TP_N as the count of test positives, in contrast to AP_N as the proportion of test positives. There were a few places elsewhere in the document where count and proportions of test positives were used loosely and these have been tidied up (see response to reviewer 1). The factor 1/|K_sample| is not needed when we are referring to counts. Apologies for the confusion this caused, and thank you for raising the issue.

“If however our prior assumptions around sensitivity are too high compared to simulation, the compensation will tend to push corrected true prevalence estimates too low (subfigure A in Fig 5). Conversely, if prior sensitivity is an overestimate, corrected true prevalence estimates will be too high (Fig 5 subfigure B). …” - The first sentence describes subfigure B, the second sentence subfigure A.

Thank you for spotting this. This is fixed.

“The accuracy of adjustment is more heavily influenced by prior assumptions of test specificity.” - Could this also have been a problem of using a too narrow prior distribution thus falsely indicating strong prior knowledge? At least, as far as I have understood, the specificity distributions you used were a lot narrower than the sensitivity distributions.

You are very probably correct, but introducing a comparison between sensitivity and specificity was a mistake on our part as they exert such different effects. We have rephrased this whole section to say “In subfigures C and D we see the accuracy of adjustment is also heavily influenced by prior assumptions of test specificity.“ We think the relative lack of certainty around sensitivity, compared to specificity, is really a reflection of the real life situation whereas there are usually many more negatives than positives. Any test must have a reasonable specificity to be useful, whereas tests which are not very sensitive are quite common.

“extreme misspecification” - I'm not sure I would call a specificity-prior of 99.5% or 99.9% instead of the true 99.75% an extreme misspecification.

This is rephrased as “systematic bias” in line with the response to your major comment. The wording of the discussion is also changed to remove the phrase misspecification.
