# Supplementary material for: Combined multiplex panel test results are a poor estimate of disease prevalence without adjustment for test error
Source: PLoS Comput Biol. 2024 Apr 26;20(4):e1012062. doi: 10.1371/journal.pcbi.1012062 (PMC11078360; doi:10.1371/journal.pcbi.1012062)
Supplement: Attachment — Submitted filename: review.docx [file pcbi.1012062.s003.docx]

The authors present statistical methods for prevalence estimation in the context of multiplex panel tests, more specifically for the case that the panel is used to test for multiple subtypes of the same pathogen from which then an overall prevalence or burden of disease is to be calculated. They show that in such a situation, small errors on the subtype test level compound and may result in significant bias of the overall prevalence estimate. The authors developed statistical methods to correct for these biases and used simulations to test their methods. They show that a counter-intuitive aspect of multiplex panel tests is that panel test sensitivity depends on component distribution, component test sensitivity and specificity, and on disease prevalence. As an example for a multiplex panel test, they use pneumococcal pneumonia that can be caused by more than 20 pneumococcal serotypes.

The paper addresses an interesting and important problem, is well structured and written, and cites relevant literature. To my knowledge the approach is novel and could be important for other researchers confronted with the task of prevalence estimation based on panel tests. For such researchers, the authors also provide tools implementing their approach in the form of a freely available R package.

I see two major issues with the paper. One is that the authors sometimes seem to contradict their own assumption of subtype independence. The second is the sensitivity of their correction methods to prior mis-specification which may question the applicability in practice. See below for more detail.

If the authors can address these issues appropriately, I would recommend the manuscript for publication.

Major issues

1. Assumption of independence of subtypes
   In the simulations in the supplementary appendix S1 (section 4), the authors seem to assume that subtypes do not occur together so that the subtype prevalences add up to a total prevalence (S1, page 6, line 6). This is in contrast to the assumption of independently occurring subtypes that the panel prevalence estimator is then derived from. Does this affect the validity of the simulations? With 20 or more serotypes, even if each of them only occurs with low prevalence, co-infection should occasionally be observed if they are independent. E.g., if we assume independence for 20 subtypes and a prevalence of 0.5% for each subtype, then the sum of the prevalences is 10%. But if we use a binomial probability for prevalence calculation (i.e. taking into account co-infection; in R: `1 – pbinom(0, 20, 0.005)`) then we get an expected (panel) prevalence of 9.54%. On the other hand, if co-infection is never observed in reality, then the serotypes may not occur independently after all, and a different statistical approach would be needed. For pneumococcal pneumonia, is it known whether different serotypes can co-occur in the same patient?
   In this context, is it correct that in Fig. 3 B and Fig. 4 the “true prevalence” is the sum of the component prevalences (thus not taking into account co-infection)? This question also applies to the panel prevalences in Fig. 5 (PCV7 to PCV20).
   In summary, the assumption of subtype independence warrants more discussion.
2. Mis-specification of priors
   As the panel prevalence adjustment method is very sensitive to the specificity of the component tests (see Fig. 4), correct prior assumptions for component sensitivity but especially specificity seem to be very important for the prevalence correction to work (see S2, Fig. 5). Although the authors acknowledge this fact (e.g., in lines 201-205), they describe that the method may fail in the case of “complete mis-specification” (line 247) or “extreme misspecification” (S2, Fig. 5 legend). But can a prior specificity of 99.5% instead of a true value of 97.5% (S2, Fig. 5 C) really be considered an extreme or even complete mis-specification? How practical can the method be with real-world data and real-world test accuracy information in the light of such sensitivity to prior mis-specification?

Minor issues

1. Because the methods (as described in the supplementary files S1 and S2) provide the foundation for the paper, it is important that the details in these files be correct. To my understanding, the mathematical notation contains several mistakes that should be corrected or clarified (see attached supplementary files with comments). Both supplementary files should be diligently proofread by the authors to ensure correctness and consistency.
2. In Fig. 2, the panels C, F, and I are not explained in the figure legend.
3. In Fig. 4, is the “component specificity” axis logarithmically scaled? This should be mentioned.
4. Fig. 5 could be improved by ordering the components not alphabetically but according to the PCV groups and then also indicating the PCV groups with horizontal brackets below the serotype labels.
5. A few typos and confusions (specificity --> sensitivity in line 217 on page 9) should be addressed (see attached manuscript file with comments).
6. I failed to install the `testerror` R package from the Bristol Vaccine Centre repository. However, installation of the development version from github worked fine.
